# Music-based interventions in rehabilitation of children and adolescents with chronic diseases: Sharing an experience from a Brazilian public hospital

**DOI:** 10.3389/fresc.2023.1116914

**Published:** 2023-03-01

**Authors:** Anna-Dulce Sales Carneiro Sampaio

**Affiliations:** Children’s Hospital, Hospital das Clínicas da Faculdade de Medicina da Universidade de São Paulo (HC-FMUSP), São Paulo, Brazil

**Keywords:** hemodialisys, chronic disease, music-based intervention, hospitalized pediatric patients, humanization, immunosuppressed patients, end-of life care, music therapy

## Abstract

Music-based interventions (MBI) in therapeutic routine have been observed as an effective tool for adjuvant therapy for patients with chronic diseases and for those with various types of disabilities. Music can serve as a pedagogical and therapeutic strategy for development of psychomotor skills in children and adolescents with long-lasting illnesses, and also as a secular way of promoting comfort and spiritual health for patients and families during difficult times; it can also be used to alleviate suffering during diagnostic and therapeutic procedures throughout the long recovery and/or rehabilitation processes. In this article, a musician and art therapist describes some simple MBI used daily in a tertiary children's hospital in Sao Paulo, where patients with chronic diseases and from lower socioeconomic background represent most of outpatients and inpatients. Some MBI developed or adapted by the author are described in detail, some of them using only the voice and others utilizing simple musical instruments, usually percussion ones, by the patients themselves. There are also reports describing MBI in special situations, such as long-lasting isolation of immunosuppressed patients (for bone marrow transplantation, for example), regular day-hospital visits for hemodialysis and religious songs for families of end-of-life or critically ill patients. In conclusion, MBI can be beneficial for improving patient experience in hospital settings, particularly for those with long-lasting or repeated stays, and can be performed in a simple and playful way, with contribution of other health professionals with some background in music, since music therapy specialists are still scarce in many countries.

## 1. Introduction

Music therapy has been observed as an effective adjuvant treatment for patients, both children and adults, with chronic diseases as well as for those with various types of disabilities (1). It has been widely recognized that music simultaneously stimulates many areas of the brain promoting brain plasticity in brain-injured patients and in children with genetic disorders that cause intellectual and physical disabilities (2, 3). Music can be useful in the evaluation, diagnosis and development of skills in patients with intellectual and/or psychomotor disabilities (4).

Music can act in the daily life of children with long-lasting diseases promoting well-being, entertainment, improvements in speech and communication, it is a pedagogical tool for special education and also an instrument for physical and cognitive rehabilitation (4, 5). Music-based interventions (MBI) in health can be adopted by different therapeutic modalities such as psychology, psychopedagogy, physiotherapy, speech therapy, occupational therapy, art therapy, music therapy, among others.

This article has the objective of presenting a detailed description of some simple musical exercises and reporting a series of experiences with music performed in a tertiary pediatric hospital (ICr-HCFMUSP) with inpatients and outpatients, mostly with chronic diseases. These MBI represent part of a broader institutional program of humanization, which includes warm welcome, ambience, various types of art therapy, schooling, religious support, nature appreciation, aimed at reducing suffering and making hospitalization, treatments and follow-up less traumatic.

At ICr-FMUSP, MBI are used in a diversity of circumstances and for different purposes, some of them are described in this article with the support of scientific literature on the subject. The references in this manuscript are composed of randomized and qualitative studies, case reports, journalistic articles and bibliographic reviews on music therapy and other musical interventions in health, with the aim of enriching the analysis of the reported experiences and the described exercises.

This text presents an account of the context of the Brazilian public health system, with its laws and forms of organization, with the view of sharing experiences with health professionals from other regions and realities, so that these activities could be adapted according to the characteristics of their patients and services.

## 2. Part 1 – different uses of music in hospital humanization

Hospitalizations, sometimes long-lasting and repeated ones, regular stays at day-hospital, visits to out-patient clinics for follow-up and diagnostic procedures represent frequent events in the life of patients with chronic conditions, and music can certainly contribute to a better experience (6).

In the Brazilian Public Health System, there is a specific law for the humanization of healthcare, establishing parameters to make processes and environments more friendly and receptive to patients, families, and health professionals (7). For pediatric patients in particular, a good experience in the hospital setting is pivotal to help the child and the family to develop a positive approach to the treatment, in thus contributing to disease control, rehabilitation and better health outcomes.

Music is largely present in our hospital routine and it is played in different settings, such as listening with earphones to selected playlists (with classical music, Brazilian popular music, songs for children and youth, among others), singing songs and playing instruments with therapists, besides regular visits of volunteer professional musicians.

### 2.1. Hemodialysis for chronic renal failure

Most children with kidney diseases leading to chronic renal failure need hemodialysis and, thus, spend many days of the week in treatment, usually in day-hospital. Hemodialysis lasts for hours and has profound impact in the routine and schooling of pediatric patients, compromising school performance and professional future. Hospital schooling - adopted as public policy in Brazil (8) and largely offered in ICr-HCFMUSP - represents an instrument for social inclusion for these patients who, being mostly neurotypical, may have a perspective of transplants and a productive adult life. Music, in addition to being officially part of Brazilian education in the field of arts, is a helpful instrument that can be used in other disciplines such as Portuguese language, mathematics, history, and special education in general, besides entertainment.

MBI are known to have the potential to decrease anxiety in adult hemodialysis patients (9). In addition to the psychological benefits, listening to music can reduce blood pressure fluctuations and other discomforts such as nausea (10); these positive effects of music are likely to be extended to pediatric patients. In ICr-HCFMUSP, the learning of musical instruments has been encouraged as, in addition to being an educational tool that stimulates memory and cognitive skills, it can be a way to relieve the boredom of hours of hemodialysis.

### 2.2. Bone marrow transplantation and other situations of long-lasting infection control isolation precautions

Most pediatric patients with cancer and other diseases associated with immunosuppression spend long-lasting periods in hospitals and therapeutic settings that require isolation precautions to avoid exogenous infections, particularly airborne transmission. Some of them undergo bone marrow transplantation and face periods of profound immunosuppression both before and after the procedure, requiring stricter measures to prevent infectious complications. During the isolation in special rooms, social interactions and physical activities are extremely limited, and thus patients develop emotional responses such as loneliness, depression, rejection, anger, anxiety, and sometimes mental confusion (6, 11). Such negative effects of repeated and long-term hospitalizations in these difficult conditions can be alleviated with some supportive measures: music and other artistic activities [drawing, painting, paper folding (origami), video] having been observed to be particularly helpful. Art therapist visits to sing and play popular songs have been well accepted and seem to induce well-being in patients and caregivers. In an intervention with cancer patients during long isolation periods in our Institution, beautiful images and sounds of Nature from the diverse Brazilian ecosystems were presented in their own rooms, in order to stimulate positive emotions (12).

It has been demonstrated that music therapy for pediatric patients in isolation can reduce pain and anxiety, promote tension release and relaxation, improve the quality of life, promote self-esteem and celebrate the positive aspects of a child's life and treatment, allowing children to express their feelings in a more normalized environment with fun age-appropriate activities (13). MBI can decrease anxiety and depression symptoms in children and adolescents with cancer, moreover it leads patients to adopt coping strategies and decreases heart rate, respiratory rate and blood pressure (14). Even during bone marrow aspirations for diagnosis, listening to music was associated to less pain (15).

The use of music and any other interventions in immunosuppressed patients require special precaution procedures and must always be previously discussed with the infection control committee.

### 2.3. Support for families with patients approaching end-of-life and critically ill ones

Music has been used in hospitals with end-of-life patients in different parts of the world and has been observed as an instrument for transforming the environment into a more peaceful place (16). Playing religious music can be used as a way to provide spiritual health to patients and families from many religions as most of them use music in their rituals.

Brazil is a lay state where people are free to choose any religion or spirituality, and there are a lot of spiritual paths; patients and families of any faith or religion need to have their spiritual health taken care of. Nourishing spirituality is one of the main concerns for adult cancer patients facing end of life (17); with children this has not been considered such a crucial issue, however for families spiritual health can be very helpful in coming to terms with the death of a beloved child.

In our pediatric intensive care unit (PICU), there is a musician, the author of this article, who has developed and compiled a repertoire of songs and prayers from the main religions present in Brazil, and is available to include songs from faiths she has not yet learned. As such, music is used to provide spiritual health in the PICU as a mean for social integration for people from minority religions, who are sometimes afraid to express their faiths fearing prejudice from other families and even from professionals.

Music therapists are often asked to care for the spiritual health of end-of-life patients with chaplains and there are ethical and training implications for this type of work (18). Sometimes people from some religions do not feel comfortable when the therapist providing spiritual care is not a cleric or not from the same religion, but oftentimes it is a matter of asking the family if they would accept that a therapist sings or plays music of their faith. Most of the families in our hospital PICU have accepted it and reported that they feel better with this kind of MBI.

## 3. Part 2 - description of some music-based exercises largely used with inpatients and outpatients

The exercises described below are as simple as singing an interactive song with a patient or playing percussion instruments in small groups; they can be useful both to assess and develop important skills for the daily lives of children in a therapeutic or a pedagogical context. The exercises can be performed individually or in small groups, with outpatients at waiting rooms, for entertainment, or in rehabilitation processes with inpatients, or in day-hospital. These exercises were adapted from ones used in music therapy (19) and, as they are so simple to perform, they could be played by other professionals with some background in music, such as nurses, occupational therapists, psychologists, social workers, and teachers as well, thus, bringing the benefits of music to health services in general, schools and child welfare institutions.

### 3.1. Interactive singing with the child

In this type of exercise, the popular song “The Old MacDonald had a farm” was adopted only as an example, and there are several interactive songs that can be used. Each culture has its own repertoire of songs for children that can be explored for therapeutic and pedagogical purposes.

Let's imagine the following situation: a therapist, preferably playing a musical instrument, sings “Old MacDonald Had a Farm” with a 6-year-old girl in rehabilitation. The professional stops the song and asks the child to mention an animal living in the farm; the patient accesses her memory with all the animals she knows and uses her cognitive skills to choose a suitable animal for a farm. Then, the child chooses a duck as an example and the song goes on, until it is interrupted again as the therapist asks the girl what sound the duck makes; the child again uses her cognitive skills and memory to mimic the duck sound and the song goes on. Soon after, the therapist asks the child again to choose another animal to put in her Old MacDonald’s Farm song and the child goes back to the same cognitive processes to choose another animal, excluding the duck.

In this simple process of singing a song in a pleasant way with a patient, the child's memory and cognition are tested in a few ways. This child may or may not have a repertoire of animals for several reasons: she may have a intellectual disability or even not have a family and cultural structure that allows her to visit a farm, watch cartoons and read books that bring animals as a reference for her repertoire of words.

This exercise can be used in different therapeutic contexts by professionals from various fields, it can provide the therapist with much information about the child's cognitive abilities and socio-cultural issues. Singing the song and interrupting it to interact with the patient is a very different gesture from listening to a recording of the song, in both situations the child is stimulated, but in different ways.

### 3.2. Interactive playing with simple musical instruments

The exercise named “I will only play my instrument if you play yours with me” takes place as follows: the therapist has an instrument and the patient has another, the professional starts playing a known melody but interrupts it in order to invite the patient to play along; every time the patient stops playing, the therapist also stops and waits until the patient starts playing it again.

This exercise is indicated for neurotypical children and also for those with impaired cognition, as it requires simple skills: first, the patient must understand that he/she has a musical instrument in hands which produces a sound, then the child must understand that music will only be produced if he/she plays the instrument; in addition, it is necessary to have an understanding that there are two people making music and that one depends on the other to play. Thus, a non-verbal relationship is established between the therapist and the child whilst this joint activity is carried out which generates music, and at the same time pleasure at performing and listening to it. This kind of exercise encourages communication and sociability in general and could be applied to patients with speaking difficulties.

In the exercise, the therapist can offer the patient a simple percussion instrument (rattle, tambourine, others), or children's melodic instruments usually tuned in the C major scale, such as a metallophone or a toy piano.

This exercise could be used with children with chronic conditions, like autism, as warming to other exercises. Music Therapy can increase quality of life and promote improvements in the total autism severity in pediatric outpatients with autism (20).

### 3.3. Group exercises: choosing and sharing musical instruments, and encouraging protagonism among children

This exercise may be performed as follows: in the center of a circle with four children there are simple percussion instruments. Each child tries out the musical instruments and finally chooses what he/she prefers to play. In this simple act of choosing an instrument, the child's protagonism is encouraged, something that is hardly ever and even allowed within the routine of treatments of patients with chronic diseases or disabilities, since everything at the hospital is mandatory. After each child chooses an instrument, with the assistance of the therapist, one is asked to start with a pulse and the others to get into the rhythm after listening to it. One patient at a time gives the initial pulse for a few moments. This is an exercise of listening, leadership and shared focus, since each child is called to start the music at a time; sociability talents are also encouraged and they may be useful in performing musical exercises and other activities in life.

This exercise can be used in group therapy and in a pedagogical context for neurotypical and disabled children. This kind of exercise is suitable for waiting rooms and is especially applicable in outpatient clinics.

Group music training can be useful to improve empathy in neurotypical children with poor social skills (21); these benefits may be also extended to children with intellectual disabilities whose sociability should be encouraged. In adults with severe learning disabilities, music group training gives them the opportunity to develop and maintain friendships (22).

## 4. Discussion

Part of the ICr-HCFMUSP experience in using musical exercises for follow-up, treatment and/or rehabilitation of children and adolescents with long-lasting diseases is presented here, inserted in a broader context of an institutional humanization program, already mentioned (7). Humanization initiatives have been fundamental to improve the experience of patients and companions in public hospitals in Brazil, and music can contribute to this field in several ways, such as reducing anxiety and even pain in medical procedures, as well as in education and in the transformation of healthcare processes of rehabilitation into more fun and effective ones.

One of the barriers that music-based interventions face in the hospital environment is the sanitation and sharing of musical instruments. Many instruments are made of wood, a material unsuitable for health facilities particularly in hospitals, where infection transmission must be strictly controlled. Plastic or metal instruments represent better options, whilst wind ones are not recommended. In light of this, some instruments must only be used by therapists and not shared with the patients; the instruments allowed to the children must be sanitized and, thus, made of perfectly sanitizable materials. Another recommendation is that, including day-hospitals, each child should have their own musical instrument.

Regarding the uses of religious songs for families with children at PICU and other wards, prejudices have been observed against people from pagan and African-Brazilian faiths. Although only 0.3% of the population declare themselves to be devotees of religions of African origin, in the city of Rio de Janeiro, 91% of cases of violent attacks linked to religious intolerance are directed to temples and people of these religions, and the public hospitals are not excluded from such behaviors (23). Therefore, hospital management and staff must be aware of cultural differences in order to avoid additional suffering for patients and their families. As Brazilian public hospitals tend to have only Christian chaplains, families from minority religions should be encouraged to request visits from their religious leaders.

Music is a very relevant aspect of Brazilian culture, being present in the daily lives of people of all ages, as it also happens in most Latin American countries. According to the Billboard charts, Brazil and Mexico are the two countries that mostly consume music on the planet (24), and the Brazilians, in particular, have the habit of listening to their own country's music (25). It is therefore easy to introduce MBI into healthcare institutions, as even children and adolescents with chronic conditions and disabilities are largely exposed to high-quality popular music. At ICr-HCFMUSP, there is a growing demand for new MBI modalities contemplating other patient groups and conditions, in addition to the ones already implemented.

In conclusion, MBI can be beneficial for improving patient experience in hospital settings, making treatments less traumatic, more effective and even pleasant. It may be beneficial particularly for patients with long-lasting or repeated stays in hospitals, and can be performed in a simple and playful way, with the contribution of other health professionals with some background in music, since music therapy specialists are still too scarce in many countries.

## Data Availability

The original contributions presented in the study are included in the article/Supplementary Material, further inquiries can be directed to the corresponding author.
